# Low-grade Appendiceal Mucinous Neoplasm in the Context of Acute Appendicitis

**DOI:** 10.7759/cureus.5159

**Published:** 2019-07-17

**Authors:** Katherine R Porter, Carlos E Ramos, Vladimir Neychev

**Affiliations:** 1 Miscellaneous, University of Central Florida College of Medicine, Orlando, USA; 2 Pathology, Health Care Corporation of America, Longwood, USA; 3 Surgery, University of Central Florida College of Medicine, Orlando, USA

**Keywords:** appendicitis, mucinous neoplasm, lamn

## Abstract

A 49-year-old woman presented with clinical signs, pre-operative imaging, and intra-operative findings suggestive of acute appendicitis. A laparoscopic appendectomy was performed. Final pathology revealed a low-grade appendiceal neoplasm with serrated architecture, and secondary acute inflammation of the appendix (5 cm in length x 0.7 cm in diameter) with a congested and hemorrhagic serosal surface. The main concern in the management of patients with low-grade appendiceal mucinous neoplasm (LAMN) is that these dysplastic tumors share such similar clinical, imaging, and intraoperative features with simple acute appendicitis, which is deemed definitively cured following surgical removal of the appendix. A definitive diagnosis of LAMN is often delayed until the final pathology report; however, this finding can have implications on the further management of the patient, especially with the risk of recurrence in situations of peritoneal dissemination or positive surgical margins. Reporting LAMN cases will increase awareness of this rare disease and will contribute to improved management in the future.

## Introduction

Appendiceal mucinous neoplasms are rare dysplastic mucinous tumors, which can be further classified as low-grade or high-grade, based on their cytologic features [1]. Low-grade appendiceal mucinous neoplasms (LAMNs) are characterized by low-grade cytologic features, a “pushing” border, substantial production of mucin, villous or flat proliferative intestinal-type mucinous epithelium, and typical confinement to the muscularis propia [2]. LAMNs are often diagnosed incidentally, as clinical presentation is rarely specific. LAMNs are non-invasive of the appendiceal epithelium, but because of their tendency to grow into the muscularis propria, they have the capacity to irritate and cause inflammation of the appendix, and can even cause the appendix to rupture [2]. These features occasionally lead to a clinical presentation consistent with acute appendicitis [2-3]. LAMNs are exceedingly rare, especially compared to the frequency of acute appendicitis, with only 1,000-2,000 cases of LAMN diagnosed in the United States each year [4]. Thus, their incidence demonstrates the importance of reporting such rare cases. We present a case of LAMN with a clinical and peri-operative presentation of simple acute appendicitis.

## Case presentation

A 49-year-old woman was admitted to the hospital for the evaluation of mild nausea and abdominal pain, which had evolved from burning, epigastric pain to aching, and right lower quadrant pain over a period of six hours. She described the pain as moderate to intense, continuous, and without alleviating or exacerbating factors. She denies anorexia, back and flank pain, and issues with bowel movements. She admitted to drinking on social occasions and revealed that she had quit smoking 20 years ago. Her past medical history was otherwise unrevealing. Her family history was significant for hypertension in her mother, and colon polyps and colon cancer in her brother and grandmother, respectively.

At initial evaluation, her vital signs were within normal limits with a temperature of 98.7ᵒF, a pulse rate 86 beats per minute, a respiratory frequency of 18 breaths per minute, and a blood pressure of 128/85. The abdominal exam revealed moderate to severe tenderness in the right lower quadrant with rebound. Normal bowel sounds on auscultation. There were no visible abdominal wall defects, and no pulsatile or pathological masses. The remainder of her physical exam was unremarkable. The laboratory evaluation was significant for an increased white blood cell count of 10.7K/mm^3^, and neutrophil count of 7.4K/mm^3^.

**Figure 1:**
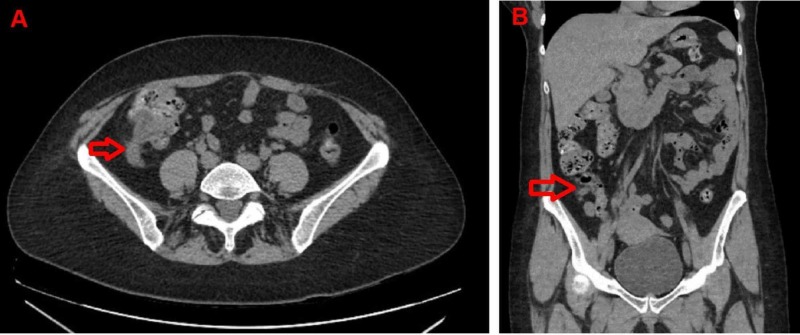
Computed tomography (CT) of the abdomen A – Axial CT scan image, and B – Coronal CT scan image. Arrows show dilated appendix measuring up to 1.1 cm with mild periappendiceal inflammatory fat stranding.

**Figure 2:**
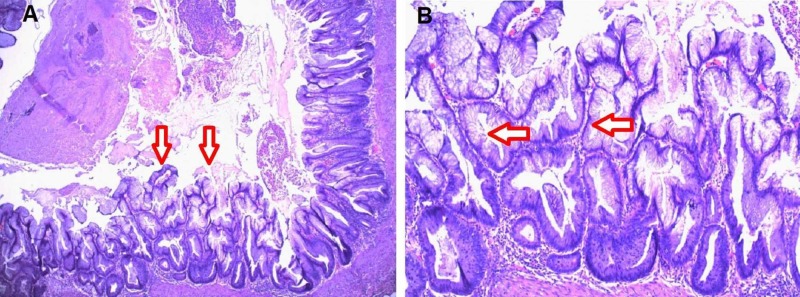
Haemotoxylin and eosin stain of the appendiceal mucosal surface A - Low power view. Arrows show low-grade appendiceal mucinous neoplasm with serrated pattern occupying the mucosal surface, exhibiting characteristic expansile growth; B - Higher power view of low-grade appendiceal mucinous neoplasm. Arrows point at dysplastic epithelium with mild cytological atypia and mucinous secretion.

Abdominal ultrasonography was inconclusive, but computed tomography (CT) of the abdomen and pelvis revealed a dilated appendix measuring up to 1.1 cm with mild inflammatory periappendiceal fat stranding and no calcification (Figures 1A-1B). Acute appendicitis was the main differential diagnosis, with the possibility of appendiceal neoplasm low on the list. A detailed discussion of the risks and benefits of a laparoscopic, possibly open, appendectomy was carried out, and informed consent was obtained. The surgery was performed without complications, and the post-operative diagnosis remained acute phlegmonous appendicitis. Surgical pathology, however, revealed a LAMN with serrated architecture, and secondary acute inflammation of the appendix (5 cm in length x 0.7 cm in diameter) with a congested and hemorrhagic serosal surface (Figures 2A-2B). Margins were free and no fecaliths were identified on sectioning.

The patient tolerated and recovered well from surgery and was discharged home on day one after surgery.

## Discussion

LAMN is rare, with only 1,000-2,000 cases being diagnosed annually in the United States [4]. Although they most often present with symptoms suggestive of acute appendicitis, appendiceal tumors are only identified in approximately 1% of all appendectomies [3,5]. Only 0.1% of all appendectomies resulted in the discovery of a primary appendiceal malignancy [5]. A low-grade serrated appendiceal mucinous neoplasm is rare, with few cases being described in the literature.

The underlying pathophysiology of this specific form of appendiceal neoplasm has not been fully elucidated. Serrated appendiceal lesions often have mutations in KRAS codons 12 and 13, but not in BRAF oncogene (mutations in BRAF are often seen in serrated colon polyps) [6-8]. However, this designation is specific to serrated polyps. Thus, without genotyping the neoplastic tissue, it would be impossible to predict the true pathophysiology of this tumor.

LAMN usually presents either asymptomatically or, as in this case, similarly to acute appendicitis - with right lower quadrant abdominal pain. Additionally, this patient’s pain progressed from epigastric diffuse pain to localized lower quadrant pain, a pathognomonic sign of acute appendicitis. The elevated white blood cell and neutrophil counts were also indicative of an acute infectious process; CT added to this clinical picture was suggestive of simple acute appendicitis. With the information available, an early appendicitis would have been virtually indistinguishable from an appendicitis secondary to appendiceal mucinous neoplasm. The overall incidence of appendicitis in the United States is approximately 1.1 to 1.8 per 1,000 population per year [9]. Thus, the majority of symptomatic appendiceal neoplasm cases are likely to be considered a primary acute appendicitis, initially.

Establishing the diagnosis of LAMN is often delayed until the final pathology results are obtained, especially when the clinical signs and the intraoperative appearance of the appendix mimic simple acute appendicitis so closely. Some report that laboratory findings of anemia and elevated tumor markers may be present in patients with LAMN, however, in the case presented, all of the typical markers for anemia - red blood cell count, hemoglobin, hematocrit, and mean corpuscular volume (MCV) - were in the normal range, and there was no indication that would have warranted or suggested testing for tumor markers [10]. The appearance of the lesion on CT is often used to distinguish between mucinous lesions like LAMNs and acute appendicitis. However, in the case presented, the CT findings were more consistent with acute appendicitis, with a dilated appendix, lack of appendiceal wall thickening and mild periappendiceal inflammatory stranding, than they were with a LAMN, which would have been indicated by the presence of eggshell appearing mural curvilinear calcifications, and the absence of such inflammatory stranding [11-13].

The differential diagnoses for LAMN also include mucinous adenocarcinoma of the appendix and high-grade appendiceal mucinous neoplasm (HAMN). Unlike LAMN, mucinous adenocarcinoma is characterized by infiltration of the appendiceal wall epithelium, often with desmoplastic reaction [2]. LAMN and HAMN are only distinguished by their degree of epithelial dysplasia [2]. The presence of low-grade cytologic features, in this case, confirmed the diagnosis of LAMN.

From a surgeon’s perspective, it is important to note that the most effective therapy for both acute appendicitis and LAMNs that are intact and confined to the appendix is an appendectomy [2]. It may present a challenge to the differential diagnosis that the patient does not present with a fever or reproducible tenderness at McBurney’s point. However, because the imaging studies demonstrated a dilated, inflamed appendix, the appendix must be removed, regardless of the cause of this inflammation. In the case of LAMN, hesitation to operate could prove catastrophic if the appendix were to rupture, spreading neoplastic cells through the peritoneum, resulting in pseudomyxoma peritonei [1].

If a LAMN was removed completely with free margins, without spilling mucin or rupturing appendix, and the pathology specimen showed no cells or mucin outside of the appendix, there is virtually no risk of recurrence, and specific follow-up or surveillance is not required.

## Conclusions

The growing number of reported cases of LAMN draws increasing awareness and contributes to a greater understanding of this disease. Distinguishing LAMN from primary acute appendicitis preoperatively remains difficult; however, it is critical that this distinction is made with post-operative pathology, as a diagnosis of LAMN carries the possibility of postoperative implications and courses of treatment not seen with primary acute appendicitis. Treatment decisions should be made through a collaboration between surgeons and pathologists and should take into account all disease-contributing factors, such as disease extent and comorbidities, in order to ensure the most favorable outcome.
